# Antibiotic Prescribing by Digital Health Care Providers as Compared to Traditional Primary Health Care Providers: Cohort Study Using Register Data

**DOI:** 10.2196/55228

**Published:** 2024-06-26

**Authors:** Andy Wallman, Kurt Svärdsudd, Kent Bobits, Thorne Wallman

**Affiliations:** 1 Department of Medical and Translational Biology Umeå University Umeå Sweden; 2 Family Medicine and Preventive Medicine Section Department of Public Health and Caring Sciences Uppsala University Uppsala Sweden; 3 Region Sörmland County Council Eskilstuna Sweden; 4 Centre for Clinical Research Sörmland Uppsala University Eskilstuna Sweden

**Keywords:** telehealth prescribing, physical-primary health care, internet-primary health care, antibiotics, prescription, infectious disease, antibiotic, prescriptions, prescribing, telehealth, health care, traditional, digital, telemedicine, virtual care, Swedish, Sweden, primary care, quality of care, online setting, ePrescription, ePrescriptions, ePrescribing, eHealth, compare, comparison, online consultation, digital care, patient record, patient records, mobile phone

## Abstract

**Background:**

“Direct-to-consumer (DTC) telemedicine” is increasing worldwide and changing the map of primary health care (PHC). Virtual care has increased in the last decade and with the ongoing COVID-19 pandemic, patients’ use of online care has increased even further. In Sweden, online consultations are a part of government-supported health care today, and there are several digital care providers on the Swedish market, which makes it possible to get in touch with a doctor within a few minutes. The fast expansion of this market has raised questions about the quality of primary care provided only in an online setting without any physical appointments. Antibiotic prescribing is a common treatment in PHC.

**Objective:**

This study aimed to compare antibiotic prescribing between digital PHC providers (internet-PHC) and traditional physical PHC providers (physical-PHC) and to determine whether prescriptions for specific diagnoses differed between internet-PHC and physical-PHC appointments, adjusted for the effects of attained age at the time of appointment, gender, and time relative to the COVID-19 pandemic.

**Methods:**

Antibiotic prescribing data based on Anatomical Therapeutic Chemical (ATC) codes were obtained for Region Sörmland residents from January 2020 until March 2021 from the Regional Administrative Office. In total, 160,238 appointments for 68,332 Sörmland residents were included (124,398 physical-PHC and 35,840 internet-PHC appointments). Prescriptions issued by internet-PHC or physical-PHC physicians were considered. Information on the appointment date, staff category serving the patient, *ICD-10* (*International Statistical Classification of Diseases, Tenth Revision*) diagnosis codes, ATC codes of prescribed medicines, and patient-attained age and gender were used.

**Results:**

A total of 160,238 health care appointments were registered, of which 18,433 led to an infection diagnosis. There were large differences in gender and attained age distributions among physical-PHC and internet-PHC appointments. Physical-PHC appointments peaked among patients aged 60-80 years while internet-PHC appointments peaked at 20-30 years of age for both genders. Antibiotics with the ATC codes J01A-J01X were prescribed in 9.3% (11,609/124,398) of physical-PHC appointments as compared with 6.1% (2201/35,840) of internet-PHC appointments. In addition, 61.3% (6412/10,454) of physical-PHC infection appointments resulted in antibiotic prescriptions, as compared with only 25.8% (2057/7979) of internet-PHC appointments. Analyses of the prescribed antibiotics showed that internet-PHC followed regional recommendations for all diagnoses. Physical-PHC also followed the recommendations but used a wider spectrum of antibiotics. The odds ratio of receiving an antibiotic prescription (after adjustments for attained age at the time of appointment, patient gender, and whether the prescription was issued before or during the COVID-19 pandemic) during an internet-PHC appointment was 0.23-0.39 as compared with a physical-PHC appointment.

**Conclusions:**

Internet-PHC appointments resulted in a significantly lower number of antibiotics prescriptions than physical-PHC appointments, adjusted for the large differences in the characteristics of patients who consult internet-PHC and physical-PHC. Internet-PHC prescribers showed appropriate prescribing according to guidelines.

## Introduction

“Digital care” is a broad term covering interactions in health care that do not require the care provider and patient to be in the same place at the same time [1]. The concept includes terms such as “telehealth,” “direct-to-consumer telemedicine,” “patient-initiated, on-demand health care with health care personnel at a distance,” and “virtual visits.” [2]

Technologies such as telehealth and online video appointments increase patients’ access to health care, for example, when transport problems, schedules, or physical disabilities make office appointments difficult. These options may save time at work or home, as well as travel time, and missed and rescheduled assignments [3]. Less travel time and waiting time at primary health care (PHC) centers mean less absence from work, which benefits both patients and their employers, but also increases health care costs for society [4].

Availability, flexibility, simplicity, and convenience attract many patients to online health care [5]. Use of the telehealth market and services also increased globally during the COVID-19 pandemic [6].

Digital care meetings are a growing part of Swedish PHC, and there are several private and public digital care providers in the Swedish market [7]. Digital care can also potentially improve access to care for residents living in the rural areas of Sweden [4,5].

Despite these positive results, prescribing antibiotics through remote consultations might be difficult because of the inability to perform physical examinations and carry out necessary testing. Given the global concern regarding antimicrobial resistance, prescribing patterns for digital care providers compared with traditional PHC are important to investigate [8].

The Strategy Group for Rational Antibiotic Use and Reduced Antibiotic Resistance (Strama) aims to counteract antibiotic resistance in Sweden by providing recommendations for the treatment of common infections [9] and has accordingly suggested quality indicators for internet-PHC prescribing for common infection diagnoses [9].

Studies have suggested that physicians among private digital care providers (internet-PHC) in Sweden are less thorough in terms of following Strama’s guidelines and recommendations than physicians practicing in traditional physical PHC (physical-PHC), and therefore prescribe antibiotics more often [10,11]. However, the results of a previous review paper showed insufficient evidence of the impact on antibiotic prescribing in primary care [8]. Studies revealed reduced antibiotic prescriptions for some infectious diagnoses by internet-PHC physicians [12,13], with others showing no differences [12-15], and specific age groups exhibiting higher antibiotic prescription rates [16]. A previous study of Swedish internet-based primary health care (PHC) showed a lower rate of prescriptions for sore throat and respiratory symptoms, with no differences observed for dysuria symptoms [12]. Another study showed that adult patients presenting with sinusitis received a higher rate of guideline-concordant diagnosis and a lower prescription of antibiotics at digital appointments as compared with in-office primary care appointments [13]. However, other studies demonstrated no difference [14,15,17,18], for example, in areas of acute respiratory infection [14] and management of urinary tract infections for most average-risk female patients, confirming that the national guidelines were followed in the majority of cases [17].

An American study showed no difference in prescribing or laboratory and imaging tests in most of the 20 studied diagnoses [18]. A randomized controlled trial demonstrated no difference and similar clinical outcomes for patients with diabetes mellitus having video compared with in-clinic consultations, as well as greater satisfaction among patients and primary care providers who used telemedicine consultations [15]. However, antibiotic prescribing was higher among children aged 0-17 years using digital appointments compared with in-office urgent care and primary care, and concordance with antibiotic prescribing guidelines was lower for digital appointments [16]. This study thus aimed to examine possible differences in antibiotic prescription patterns between internet-PHC and physical-PHC physicians in a sufficiently large study population to provide high statistical power, taking account of the effects of patient-attained age, gender, and time relative to the COVID-19 pandemic.

## Methods

### Setting

All Swedish residents have a personal identification number, which includes their date of birth and gender, and this is used for identification in all official registers in the country. Moreover, all residents are covered by the National Health Insurance Act, which allows them to see any physician they choose at heavily reduced fees [19].

Drugs may be prescribed only by licensed physicians and by midwives and nurses who meet specific requirements [20]. All medical care in Sweden is organized within regions, geographically corresponding to counties. PHC (public or private), including physical-PHC or internet-PHC, is usually the first point of call for patients because of its accessibility [21].

Internet-PHCs were established in Sweden in 2016, including services allowing patients to access health care using a smartphone or computer [4]. The 4 largest digital health care companies in Region Sörmland have approximately 80% of the collective digital market share in the region. The Swedish digital care system is designed to work as part of the Swedish welfare system, which means that the region reimburses the main costs for health care, prescribed drugs, and also for internet-PHC.

### Ethical Considerations

The study was carried out in accordance with the tenets of the Declaration of Helsinki and the Swedish Ethical Review Authority (diary number 2020-04098) approved the study protocol. The previous local ethics committees have been closed down and since the 1990s have been replaced by the Ethical Review Authority, a government agent checking that ethics applications follow the law of research ethics. Data were collected in standard procedures in the Region Sörmland Administrative Office for financial reimbursement to PHC caregivers and quality assurance of PHC provided within the region. The approved study protocol allowed for analyses of these collected data.

### Data Sources

Data for all appointments with physical-PHC and the 4 largest internet-PHCs in Region Sörmland from January 1, 2020, until March 31, 2021, irrespective of residency in Sweden, were obtained from the Region Sörmland Administrative Office. Data were collected in standard reports from the caregivers operating in the region. The data set included 3,064,680 appointment records for 1,238,183 patients, including 1,482,931 appointment records for 785,583 Sörmland and non-Sörmland residents consulting physical-PHC or the 4 largest internet-PHCs. Out of this material, the main study population for this report was 160,238 appointments for 68,332 Sörmland residents consulting regional physical-PHC or the 4 largest internet-PHCs. Information on the size of the Region Sörmland population by age and gender was obtained from the Swedish government agency Statistics Sweden. Data were analyzed using the Statistical Analysis System (SAS; version 9.3) [22].

Variables obtained for each appointment included the patient’s personal identification number, which provided information on gender and attained age, residential area code, care provider (physical-PHC or internet-PHC), appointment date, diagnosis according to the *ICD-10* (*International Statistical Classification of Diseases, Tenth Revision*) [23], prescriptions according to Anatomical Therapeutic Chemical code [24], and date with respect to the COVID-19 pandemic. The data set was complete with no missing data.

### Statistical Analysis

Simple differences between continuous variables were tested with Student *t* test and differences between categorical variables with chi-square tests. The effects of care provider (physical-PHC or internet-PHC) on antibiotic treatments for 6 diagnoses according to Strama [25], adjusted for the effects of attained age at the time of appointment, gender, and pandemic period, were analyzed by descending logistic regression analysis (probability modeled as the presence of antibiotic treatment). The time in relation to the pandemic period (before or during) was defined as 0 (before March 1, 2020) and 1 (from March 1, 2020, onwards).

Statistical power was computed using the SAS power procedure. The statistical power of the study was >99.9%. The SAS logistic regression procedure provided information on estimates and their 95% CIs, Wald chi-square (test parameter), odds ratios and their 95% CIs, the concordance index C (a measure of explanation similar to the receiver operating characteristic curve), and *P* values. A large number of statistical tests were performed, and Bonferroni adjustment was made to avoid mass significance issues. *P*<.005 was therefore used to indicate statistical significance.

## Results

Table 1 shows the entire data set population for Sörmland and non-Sörmland residents according to age and gender. Non-Sörmland patients accounted for the vast majority of internet-PHC appointments.

Table 2 shows the total and mean numbers of appointments among Sörmland residents according to age and gender. Women accounted for more appointments than men in physical-PHC as well as internet-PHC appointments, and overall, there were more physical-PHC than internet-PHC appointments. The average number of appointments per patient was slightly higher among physical-PHC consultations.

**Figure 1:**
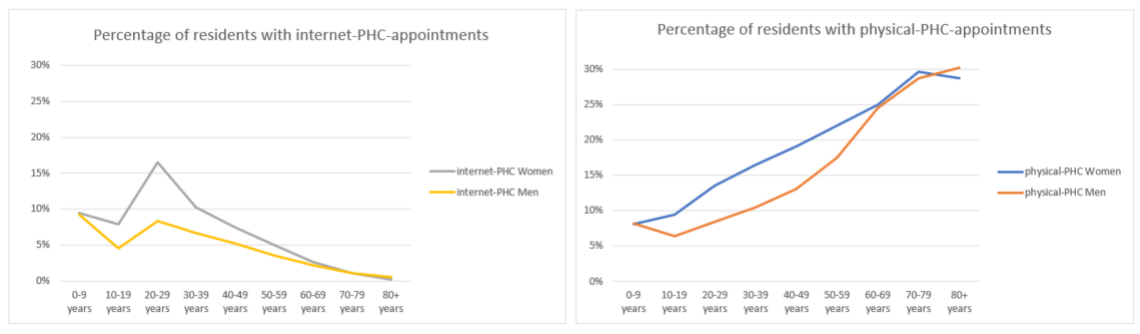
Patient age and gender distributions among physical-primary health care and internet-primary health care appointments. PHC: primary health care.

Figure 1 shows the gender and attained age distributions among physical-PHC and internet-PHC appointments. The levels of physical-PHC appointments peaked among patients aged 60-80 years for both genders, while internet-PHC appointments peaked at 20-30 years of age for both genders.

Antibiotics with the Anatomical Therapeutic Chemical codes J01A-J01X were prescribed for all purposes during 11,609 of 124,398 (9.3%) physical-PHC appointments (Table 3) as compared with 2201 of 35,840 (6.14%) internet-PHC appointments. The most common class of antibiotics prescribed was J01C for both men and women. Patients were prescribed antibiotics in J01 Groups D, E, and F less frequently during internet-PHC appointments, while patients with physical-PHC appointments were exposed to a broader prescription pattern. Appointments leading to antibiotic prescriptions were more frequent before the COVID-19 pandemic than during the pandemic.

**Table 1:** Characteristics of the study population (age and gender distribution) in the data set examined over a period of 15 months (from January 1, 2020, to March 31, 2021). Data for all physical-primary health care providers in the Region of Sörmland and all visits to the 4 largest internet-primary health care providers in Sweden.

Characteristics		Sörmland residents				Non-Sörmland residents	
		Sörmland population	Physical-PHC^a^	Internet-PHC^b^	Physical-PHC		Internet-PHC
**Age of women (years), n (%)**							
	0-9	17,123 (11.5)	1384 (5)	1621 (15.8)	67 (6.1)		57,447 (13.4)
	10-19	17,536 (11.8)	1654 (5.9)	1383 (13.5)	106 (9.7)		51,282 (12.0)
	20-29	15,524 (10.4)	2090 (7.5)	2555 (24.9)	193 (17.7)		114,504 (26.8)
	30-39	17,672 (11.9)	2905 (10.4)	1808 (17.6)	160 (14.7)		81,873 (19.2)
	40-49	17,640 (11.8)	3365 (12.6)	1319 (12.8)	115 (10.5)		57,027 (13.4)
	50-59	18,799 (12.6)	4142 (14.8)	938 (9.1)	155 (14.2)		39,209 (9.2)
	60-69	17,245 (11.6)	4298 (15.4)	444 (4.3)	143 (13.1)		17,906 (4.2)
	70-79	17,343 (11.6)	5137 (18.4)	185 (1.8)	104 (9.5)		6903 (1.6)
	80+	10,226 (6.9)	2932 (10.5)	20 (0.2)	49 (4.5)		1128 (0.3)
Total, n (%)		149,108 (100)	27,907 (55)	10,273 (58.4)	1092 (53.6)		427,279 (59.7)
**Age of men (years), n (%)**							
	0-9	18,226 (12.1)	1498 (6.6)	1679 (22.9)	55 (5.8)		58,049 (20.2)
	10-19	18,823 (12.5)	1191 (5.2)	856 (11.7)	69 (7.3)		31,649 (11)
	20-29	17,070 (11.4)	1426 (6.3)	1422 (19.4)	133 (14)		59,267 (20.6)
	30-39	18,103 (12.9)	1895 (8.3)	1199 (16.4)	138 (14.6)		51,902 (18)
	40-49	17,894 (11.9)	2326 (10.2)	930 (12.7)	127 (13.4)		39,050 (13.6)
	50-59	19,350 (12.9)	3391 (14.9)	679 (9.3)	124 (13.1)		27,395 (9.5)
	60-69	16,916 (11.3)	4146 (18.2)	364 (5)	142 (15)		13,920 (4.8)
	70-79	16,421 (10.9)	4705 (20.6)	167 (2.3)	128 (13.5)		5,755 (2)
	80+	7418 (5)	2242 (9.8)	36 (0.5)	31 (3.3)		946 (0.3)
Total, n (%)		150,221 (100)	22,820 (45)	7332 (41.6)	947 (46.4)		287,933 (40.3)

^a^Physical-PHC: number of Sörmland and non-Sörmland residents using Sörmland-based PHC.

^b^Internet-PHC: number of Sörmland and non-Sörmland residents using-based PHC.

**Table 2:** Number of appointments to primary health care among Sörmland residents.

Number of appointments			Physical-PHC^a^		Internet-PHC^b^		*P* value
**Women, n (%)**			69,176 (75.7)		22,270 (24.4)		<.001
	Average, n	2.72		2.53		<.001	
	IQR	1-3		1-3		—^c^	
**Men, n (%)**			55,222 (80.3)		13,570 (19.7)		<.001
	Average, n	2.64		2.21		<.001	
	IQR	1-3		1-2		—	

^a^Physical-PHC: Sörmland-based primary health care.

^b^Internet-PHC: internet-based primary health care.

^c^Not applicable.

**Table 3:** Antibiotic prescriptions among Sörmland residents. All gender-specific differences between physical-primary health care and internet-primary health care, as well as those in the period between before and during the COVID-19 pandemic, were significant (*P*<.001).

Prescriptions				Physical-PHC^a^, n (%)					Internet-PHC^b^, n (%)		
				Women		Men		Women			Men
**Antibiotics (ATC^c^)**											
	J01A^d^		671 (1)		467 (0.9)		215 (1)			94 (0.7)	
	J01C^e^		4804 (6.9)		3211 (5.8)		1008 (4.5)			258 (1.9)	
	J01D^f^		43 (0.06)		29 (0.05)		0 (0)			3 (0.02)	
	J01E^g^		130 (0.2)		39 (0.07)		5 (0.02)			0 (0)	
	J01F^h^		339 (0.05)		248 (0.5)		30 (0.1)			20 (0.2)	
	J01M^i^		183 (0.3)		300 (0.5)		4 (0.02)			3 (0.02)	
	J01X^j^		1080 (1.6)		213 (0.4)		562 (2.5)			6 (0.04)	
**COVID-19 pandemic**											
		Before the pandemic	1338 (11.5)		912 (9.7)		132 (8.4)			22 (1.7)	
		During the pandemic	5793 (10.1)		3566 (7.8)		1685 (6.8)			362 (3)	
		Total	7131 (10.3)		4478 (8.1)		1817 (8.2)			384 (2.8)	

^a^Physical-PHC: Sörmland primary health care.

^b^Internet-PHC: internet-based primary health care.

^c^ATC: Anatomical Therapeutic Chemical.

^d^J01A: tetracyclines.

^e^J01C: beta-lactamases.

^f^J01D: other beta-lactamases.

^g^J01E: sulfonamides.

^h^J01F: macrolides.

^i^J01M: quinolones.

^j^J01X: other antibacterial agents.

The total number of appointments with Strama-defined infection diagnoses was 18,433/160,238 (11.5%; Table 4). Physical-PHC appointments led to an infection diagnosis in 8.4% of cases compared with 22.3% of internet-PHC appointments. Appointments resulting in infection diagnoses were more frequent for internet-PHC (7979/35,840, 22.3%) compared with physical-PHC (10,454/124,398, 8.4%), and more frequent before the COVID-19 pandemic for both providers.

**Table 4:** Number and proportions of appointments for Sörmland residents with infectious diseases according to Strama antibiotic guidelines in Sweden. All gender-specific differences between physical-primary health care and internet-primary health as well as the period between before and during the COVID-19 pandemic were significant (*P*<.001).

Infections		Physical-PHC^a^, n (%)				Internet-PHC^b^, n (%)		
		Women	Men	Total	Women		Men	Total
Acne		305 (0.44)	170 (0.31)	475 (0.38)	839 (3.77)		342 (2.52)	1181 (3.3)
Acute bronchitis		291 (0.42)	207 (0.37)	498 (0.4)	56 (0.25)		31 (0.23)	87 (0.24)
Acute cystitis		1869 (2.7)	419 (0.76)	2288 (1.84)	1451 (6.52)		21 (0.15)	1472 (4.11)
Acute otitis media		402 (0.58)	402 (0.73)	804 (0.65)	13 (0.06)		14 (0.1)	27 (0.08)
Acute rhinosinusitis		299 (0.43)	126 (0.23)	425 (0.34)	212 (0.95)		82 (0.6)	294 (0.82)
Lyme borreliosis		235 (0.34)	180 (0.35)	415 (0.33)	116 (0.52)		110 (0.81)	226 (0.63)
Erysipelas		139 (0.2)	170 (0.31)	309 (0.25)	12 (0.05)		12 (0.09)	24 (0.07)
Pharyngotonsillitis		432 (0.62)	264 (0.48)	696 (0.56)	469 (2.11)		216 (1.59)	685 (1.91)
Cough		727 (1.05)	617 (1.12)	1344 (1.08)	456 (2.05)		366 (2.7)	822 (2.29)
Impetigo		86 (0.12)	73 (0.13)	159 (0.13)	177 (0.79)		150 (1.11)	327 (0.91)
Carbuncle, furuncle, etc		128 (0.19)	142 (0.26)	270 (0.22)	35 (0.16)		32 (0.24)	67 (0.19)
Chlamydia infection^c^		3 (0)	7 (0.01)	10 (0.01)	26 (0.12)		26 (0.19)	52 (0.15)
Genital mycoplasma^c^		2 (0)	1 (0)	3 (0)	2 (0.01)		8 (0.06)	10 (0.03)
Ingrown nail infection		67 (0.1)	77 (0.14)	144 (0.12)	39 (0.18)		45 (0.33)	84 (0.23)
Unspecified skin infection		399 (0.58)	373 (0.68)	772 (0.62)	212 (0.95)		146 (1.08)	358 (1)
Pneumonia		152 (0.22)	116 (0.21)	268 (0.22)	2 (0.01)		3 (0.02)	5 (0.01)
Upper respiratory tract infection		931 (1.35)	643 (1.16)	1574 (1.27)	1419 (6.37)		969 (7.14)	2388 (6.66)
**COVID-19 pandemic**								
	Before COVID-19 pandemic	1565 (13.48)	1064 (11.32)	2629 (12.51)	558 (25.82)		290 (22.41)	848 (25.54)
	During COVID-19 pandemic	4902 (8.51)	2923 (6.38)	7825 (7.57)	4893 (24.33)		2238 (18.23)	7131 (22.02)
	Total	6467 (9.35)	3987 (7.22)	10,454 (8.4)	5451 (24.48)		2,528 (18.63)	7979 (22.26)

^a^Physical-PHC: Sörmland primary health care.

^b^Internet-PHC: internet-based primary health care.

^c^Patients not treated in PHC in Region Sörmland.

A total of 6412 (61.3%/10,454) physical-PHC appointments for patients with Strama-defined infection diagnoses resulted in antibiotic prescriptions, as compared with 2057 (of 7979, 25.8%) for internet-PHC appointments (Table 5). For all diagnoses, antibiotic prescriptions were lower for internet-PHC appointments. Internet-PHC prescribing was approximately at Strama-recommended levels (<5%) for bronchitis, cough, and upper respiratory tract infections (no Strama goals were available for cystitis, chlamydia, and mycoplasma infections), but higher than recommended for other diagnoses (8.3%-37% of diagnoses).

Analyses of the prescribed antibiotics showed that internet-PHC followed regional recommendations for all diagnoses (Multimedia Appendix 1), while physical-PHC also followed the recommendations, but used a wider spectrum of antibiotics.

Logistic regression analyses (Table S1 in Multimedia Appendix 2) of medication in relation to disease showed that prescriptions of antibiotics for internet-PHC were still lower (OR 0.23-0.39) than those for physical-PHC for the 6 main diagnoses leading to antibiotic prescriptions, even after adjustments for attained age at the time of appointment, patient sex, and whether the prescription was issued before or during the COVID-19 pandemic. J01C prescribing for acute cystitis showed the lowest OR of 0.23, and the highest OR was shown for J01A prescribing for acne diagnosis.

**Table 5:** Antibiotic prescriptions in Anatomical Therapeutic Chemical groups J01A-J01X for infectious diseases in relation to the number of patients with the disease among Sörmland residents. All differences between physical-primary health care and internet-primary health care were significant (*P*<.001).

Infections	Treatment with any of J01A-J01X	
	Regional-based PHC^a^	Internet-based PHC
Acne	185 (39)	212 (18)
Acute bronchitis	146 (29.3)	2 (2.3)
Acute cystitis	2235 (97.7)	1209 (82.1)
Acute otitis media	727 (90.4)	10 (37)
Acute rhinosinusitis	274 (64.5)	29 (9.9)
Lyme borreliosis	408 (98.3)	194 (85.8)
Erysipelas	289 (93.5)	11 (45.8)
Pharyngotonsillitis	573 (82.3)	214 (31.2)
Cough	76 (5.7)	0 (0)
Impetigo	96 (60.4)	63 (19.3)
Carbuncle, furuncle, etc	222 (82.2)	9 (13.4)
Chlamydia infection^b^	10 (100)	15 (28.9)
Genital mycoplasma^b^	3 (100)	8 (80)
Ingrown nail infection	120 (83.3)	7 (8.3)
Unspecified skin infection	653 (84.6)	59 (16.5)
Pneumonia	239 (89.2)	1 (20)
Upper respiratory tract infection	156 (9.9)	14 (0.6)
Total	6412 (61.3)	2057 (25.8)

^a^PHC: primary health care.

^b^Patients not treated in PHC in Region Sörmland.

## Discussion

### Main Findings

The results of this study showed that the patients differ in age and gender between internet-PHC and physical-PHC. Infection diagnoses were more common in internet-PHC (22.3%, 7979/35,840 of appointments) than physical-PHC (8.4%, 10,454/124,398) but antibiotics were prescribed in less degree for infection diagnoses appointments, internet-PHC, 25.8% (2057/7979) versus 61.3% (6412/10,454) for physical-PHC appointments. On average, internet-PHC providers prescribed antibiotics for 6 infection diagnoses 70% less often than physical-PHC providers (OR 0.23-0.39), after taking account of the effects of differences in gender and age distributions between providers, and the period in relation to the COVID-19 pandemic. The results were similar for all 6 diagnoses tested in multivariate analysis.

### Study Strengths and Limitations

The strengths of this study included the use of data that were representative of a defined geographical area, thus minimizing the risk of selection bias regarding the included population. Moreover, the study population was large enough to avoid problems associated with low statistical power. Because the outcome variables were dichotomous, logistic regression was used to obtain the main results. The study limitations included the lack of access to medical record data. However, there was no reason to believe that the registration of diagnoses and treatments would be biased to an extent that would affect the results.

Prescribing of antibiotics in Sweden decreased by more than a quarter over the past 10 years [26], and strategies and goals to decrease antibiotic use are also set globally to decrease antimicrobial resistance.

These results indicated that infection diagnoses were much more frequent in internet-PHC appointments (22.3% [7979/35,840] of all appointments) compared with physical-PHC (8.4% [10,454/124,398]). This might be because these diagnoses are more common among younger people. Most patients chose internet-PHC for acne, impetigo, acute cystitis, and upper respiratory diseases. This might be an age effect, but these are also diagnoses that can be made by medical history or video observations. The high number of upper respiratory disease diagnoses may also be related to the COVID-19 pandemic.

Considering the effect of the pandemic, the degree of infection diagnoses by physical-PHC dropped from 12.7% to 7.6%, compared with 25.5% to 22% for internet-PHC. Hygiene recommendations probably decreased the transmission of common infections and physical appointments [27], with patients with an infection diagnosis shifting from physical-PHC before the pandemic (2629 physical- vs 848 internet-based) to internet-PHC during the pandemic (7825 physical vs 7131 internet appointments).

This study demonstrated that internet-PHC providers prescribed fewer antibiotics than physical-PHC providers during the study period, in accordance with earlier, smaller studies in Sweden [12], but in contrast to reports in the Swedish daily press [10]. The higher and broader pattern of antibiotic prescriptions in physical-PHC is probably related to the broader range of diagnoses and possibly to laboratory test results and more complicated or multiple diseases [25].

Antibiotic prescribing levels for defined infectious diseases were significantly lower for internet-PHC for all 17 Strama [25] defined infections studied. Various respiratory infections were rarely treated with antibiotics by internet-PHC, following Strama’s recommendations (<5%), despite the fact that the antibiotic prescription limit is not always possible or likely to be met [25].

Infection diagnoses often need to be confirmed by laboratory tests before prescribing, leading to less prescribing in internet-PHC. Diagnoses for which antibiotics were frequently prescribed included acute cystitis (82.1% of diagnosed) and Lyme borreliosis (85.5%), which was in line with Strama recommendations [25] for internet-PHCs. Patients with these diagnoses were prescribed antibiotics more frequently in physical-PHC.

Uncomplicated acute cystitis can be diagnosed only with an online appointment and treated with the first-line antibiotics nitrofurantoin and pivmecillinam, without laboratory testing, using internet-PHC. This appeared to be the case for other infections as well as with a higher degree of prescribing for visually diagnosable infections, such as skin infections and acne. Patients may also initiate contact with internet-PHC providers to change the administration formula after earlier contact with a physical-PHC provider. Further studies are therefore needed to establish the timing for those who make the first diagnosis and when antibiotics are prescribed to determine if the recommended Strama <5% level is adequate.

Internet appointments have several advantages, of which the most important is quick access. Internet appointments save time for the patients, are comfortable, suitable, and seem to be sufficient for making simpler diagnoses and issuing prescriptions. Internet-PHC appointments increased during the COVID-19 pandemic to avoid exposure to other patients in waiting rooms or in-person consultations. In Sweden, prescribing is almost totally digitalized, making internet-PHC prescribing easier, with patients receiving their prescriptions online instantly.

In addition to the above advantages of internet-PHC, it may have disadvantages, including leading to more appointments for symptoms that would not lead to a physical-PHC appointment. Younger patients and women tend to use internet-PHC more often due to a higher degree of digital literacy, whereas the technical threshold may exclude elderly and disabled patients. Further studies should determine if an increase in internet-PHC use releases physical-PHC resources for these groups. Internet-based PHC may fill a gap in the health care system and physical-PHC and internet-PHC may complement each other.

### Conclusions

This study demonstrated substantial differences in the characteristics of patients who consult internet-PHC and physical-PHC providers in Sweden. The proportion of patients who chose internet-PHC increased during the COVID-19 pandemic, but the rate of infection diagnoses decreased in internet-PHC. The probability of a patient with an infection diagnosis receiving antibiotics after an internet-PHC visit was reduced compared with physical-PHC, after adjusting for attained age at the time of appointment, gender, and the pandemic. Internet physicians thus prescribe fewer antibiotics than physicians in physical consultations and show appropriate prescribing according to the recommendations.

## Appendix

Multimedia Appendix 1 Prescriptions for infectious diseases in relation to the number of patients with the disease among Sörmland residents.

Multimedia Appendix 2 Logistic regression analyses of antibiotics for the most common diagnoses in relation to disease, regional- versus internet-based primary health care, age at the time of appointment, and prescription before or during the COVID-19 pandemic.

## Abbreviations

ATC: Anatomical Therapeutic Chemical
DTC: direct-to-consumer
ICD-10: International Statistical Classification of Diseases, Tenth Revision
PHC: primary health care
SAS: Statistical Analysis System
Strama: Strategy Group for Rational Antibiotic Use and Reduced Antibiotic Resistance

## Data Availability

The data sets generated during and/or analyzed during this study are not publicly available due to ethical approval restraints regarding patients’ personal health data but are available from the corresponding author on reasonable request.
