# Supplementary material for: Antibiotic Prescribing by Digital Health Care Providers as Compared to Traditional Primary Health Care Providers: Cohort Study Using Register Data
Source: J Med Internet Res. 2024 Jun 26;26:e55228. doi: 10.2196/55228 (PMC11237768; doi:10.2196/55228)
Supplement: Multimedia Appendix 1 [file jmir_v26i1e55228_app1.docx]

Supplementary Table 5a.

|  | Physical-PHC | | | | | | | Internet-PHC | | | | | | |
| --- | --- | --- | --- | --- | --- | --- | --- | --- | --- | --- | --- | --- | --- | --- |
| Diagnosis | J01A | J01C | J01D | J01E | J01F | J01M | J01X | J01A | J01C | J01D | J01E | J01F | J01M | J01X |
| Acne | 178 (37.5) | 7 (1.5) | 0 (0) | 0 (0) | 0 (0) | 0 (0) | 0 (0) | 212 (18.0) | 0 (0) | 0 (0) | 0 (0) | 0 (0) | 0 (0) | 0 (0) |
| Acute bronchitis | 79 (15.9) | 57 (11.5) | 0 (0) | 0 (0) | 8 (1.6) | 2 (0.4) | 1 (0.2) | 2 (2.3) | 0 (0) | 0 (0) | 0 (0) | 0 (0) | 0 (0) | 0 (0) |
| Acute cystitis | 3 (0.1) | 1241 (54.2) | 21 (0.9) | 77 (3.4) | 2 (0.1) | 179 (7.8) | 742 (32.4) | 0 (0) | 645 (43.8) | 0 (0) | 4 (0.3) | 1 (0.1) | 6 (0.4) | 560 (38.0) |
| Acute otitis media | 2 (0.3) | 710 (88.3) | 0 (0) | 2 (0.3) | 13 (1.6) | 2 (0.3) | 0 (0) | 0 (0) | 10 (37.0) | 0 (0) | 0 (0) | 0 (0) | 0 (0) | 0 (0) |
| Acute rhinosinusitis | 72 (16.9) | 196 (46.1) | 0 (0) | 0 (0) | 2 (1.2) | 1 (0.2) | 0 (0) | 4 (1.4) | 25 (8.5) | 0 (0) | 0 (0) | 0 (0) | 0 (0) | 0 (0) |
| Lyme borreliosis | 62 (14.9) | 346 (83.4) | 0 (0) | 0 (0) | 1 (0.2) | 0 (0) | 0 (0) | 8 (3.5) | 185 (81.9) | 0 (0) | 0 (0) | 1 (0.4) | 0 (0) | 0 (0) |
| Erysipelas | 0 (0) | 240 (77.7) | 4 (1.3) | 0 (0) | 46 (14.9) | 1 (0.3) | 0 (0) | 0 (0) | 8 (33.3) | 0 (0) | 0 (0) | 3 (12.5) | 0 (0) | 0 (0) |
| Pharyngotonsillitis | 2 (0.3) | 520 (74.7) | 8 (1.2) | 0 (0) | 46 (6.6) | 0 (0) | 0 (0) | 0 (0) | 196 (28.6) | 0 (0) | 0 (0) | 18 (2.6) | 0 (0) | 0 (0) |
| Cough | 24 (1.8) | 38 (2.8) | 0 (0) | 1 (0.1) | 10 (0.7) | 2 (0.2) | 1 (0.1) | 0 (0) | 0 (0) | 0 (0) | 0 (0) | 0 (0) | 0 (0) | 0 (0) |
| Impetigo | 0 (0) | 88 (55.4) | 7 (4.4) | 0 (0) | 4 (2.5) | 0 (0) | 0 (0) | 0 (0) | 56 (17.1) | 2 (0.6) | 0 (0) | 5 (1.5) | 0 (0) | 0 (0) |
| Carbuncle, furuncle, etc. | 3 (1.1) | 183 (67.8) | 1 (0.4) | 1 (0.4) | 33 (12.2) | 1 (0.4) | 1 (0.4) | 0 (0) | 9 (13.4) | 0 (0) | 0 (0) | 0 (0) | 0 (0) | 0 (0) |
| Chlamydia infection* | 10 (100.0) | 0 (0) | 0 (0) | 0 (0) | 0 (0) | 0 (0) | 0 (0) | 15 (28.9) | 0 (0) | 0 (0) | 0 (0) | 0 (0) | 0 (0) | 0 (0) |
| Genital mycoplasma* | 1 (33.3) | 0 (0) | 0 (0) | 0 (0) | 1 (33.3) | 1 (33.3) | 0 (0) | 0 (0) | 0 (0) | 0 (0) | 0 (0) | 8 (80.0) | 0 (0) | 0 (0) |
| Ingrown nail infection | 0 (0) | 109 (75.7) | 0 (0) | 0 (0) | 12 (8.3) | 1 (0.7) | 0 (0) | 0 (0) | 7 (8.3) | 0 (0) | 0 (0) | 0 (0) | 0 (0) | 0 (0) |
| Unspecified skin infection | 2 (0.3) | 583 (75.5) | 2 (0.3) | 0 (0) | 64 (8.3) | 3 (0.4) | 0 (0) | 0 (0) | 54 (15.1) | 0 (0) | 0 (0) | 5 (1.4) | 0 (0) | 0 (0) |
| Pneumonia | 67 (25.0) | 165 (61.6) | 0 (0) | 1 (0.4) | 6 (2.2) | 3 (1.3) | 0 (0) | 1 (20.0) | 0 (0) | 0 (0) | 0 (0) | 0 (0) | 0 (0) | 0 (0) |
| Upper respiratory tract infection | 41 (2.6) | 103 (6.5) | 0 (0) | 0 (0) | 8 (0.5) | 2 (0.1) | 2 (0.1) | 1 (0.04) | 1 (0.5) | 1 (0.04) | 0 (0) | 1 (0.04) | 0 (0) | 0 (0) |
| Total | 546 (48.0) | 4586 (57.2) | 43 (59.7) | 82 (48.5) | 259 (44.1) | 198 (41.0) | 747 (57.8) | 243 (78.6) | 1203 (95.0) | 3 (100) | 4 (80.0) | 42 (84.0) | 6 (85.7) | 560 (98.6) |

Values given as n (%).).* patients not treated in PHC in Region Sörmland
