# Supplementary material for: Antibiotic Prescribing by Digital Health Care Providers as Compared to Traditional Primary Health Care Providers: Cohort Study Using Register Data
Source: J Med Internet Res. 2024 Jun 26;26:e55228. doi: 10.2196/55228 (PMC11237768; doi:10.2196/55228)
Supplement: Multimedia Appendix 2 [file jmir_v26i1e55228_app2.docx]

**Table S1**. Logistic regression analyses of antibiotics for the most common diagnoses in relation to disease, regional- versus internet-based primary health care, age at the time of appointment, and prescription before or during the COVID-19 pandemic.

| Medication for disease | Parameter | Estimate | SE^a^ | Wald *χ*^2^ | OR^b^ (95% CI) | *P* value | C-index^c^ |
| --- | --- | --- | --- | --- | --- | --- | --- |
| J01A-acne | Care | −0.9473 | 0.0836 | 128.4 | 0.39 (0.33-0.46) | <.001 | 0.71 |
|  | Age | 0.0017 | 0.0014 | 1.5 | 1 (0.999-1.004) | .22 |  |
|  | Gender | −0.0930 | 0.0564 | 2.7 | 0.91 (0.82-1.02) | .10 |  |
|  | Pandemic | −0.5988 | 0.0663 | 81.5 | 0.55 (0.48-0.63) | <.001 |  |
| J01C-acute cystitis | Care | −1.4604 | 0.0359 | 1653.3 | 0.23 (0.22-0.25) | <.001 | 0.72 |
|  | Age | −0−0212 | 0.0005 | 1805.9 | 0.98 (0.98-0.98) | <.001 |  |
|  | Gender | −0.0033 | 0.0236 | 0.02 | 1 (0.95-1.04) | .89 |  |
|  | Pandemic | −0.1574 | 0.0299 | 27.7 | 0.85 (0.81-0.91) | <.001 |  |
| J01C-Lyme  borreliosis | Care | −1.2690 | 0.0347 | 1339.9 | 0.28 (0.26-0.30) | <.001 | 0.66 |
|  | Age | −0.0207 | 0.0005 | 1823.8 | 0.98 (0.98-0.98) | <.001 |  |
|  | Gender | −0.2706 | 0.0228 | 140.5 | 0.76 (0.73-0.80) | <.001 |  |
|  | Pandemic | −0.1704 | 0.0288 | 34.9 | 0.84 (0.80-0.89) | <.001 |  |
| J01C-pharyngotonsillitis | Care | −1.2615 | 0.0347 | 1324.9 | 0.28 (0.27-0.30) | <.001 | 0.65 |
|  | Age | −0.0177 | 0.0005 | 1319.5 | 0.98 (0.98-0.98) | <.001 |  |
|  | Gender | −0.2433 | 0.0226 | 115.4 | 0.78 (0.75-0.82) | <.001 |  |
|  | Pandemic | −0.0822 | 0.0293 | 7.9 | 0.92 (0.87-0.98) | .005 |  |
| J01C-impetigo | Care | −1.1839 | 0.0335 | 1252.6 | 0.31 (0.29-0.33) | <.001 | 0.63 |
|  | Age | −0.0193 | 0.0005 | 1639.5 | 0.98 (0.98-0.98) | <.001 |  |
|  | Gender | −0.2552 | 0.0223 | 130.9 | 0.78 (0.74-0.81) | <.001 |  |
|  | Pandemic | −0.1169 | 0.0286 | 16.7 | 0.89 (0.84-0.94) | <.001 |  |
| J01C-unspecified  skin infection | Care | −1.2144 | 0.0341 | 1269.7 | 0.30 (0.28-0.32) | <.001 | 0.65 |
|  | Age | −0.0195 | 0.0005 | 1620.8 | 0.98 (0.98-0.98) | <.001 |  |
|  | Gender | −0.2728 | 0.0227 | 144.5 | 0.76 (0.73-0.80) | <.001 |  |
|  | Pandemic | −0.1444 | 0.0289 | 24.9 | 0.87 (0.82-0.92) | <.001 |  |

^a^SE: standard error.

^b^OR: odds ratio.

^c^C-index: Concordance index (the measure of degree to which medication for a disease may be explained by parameters).
